# Role of Rho Kinase in Microvascular Damage Following Cerebral Ischemia Reperfusion in Rats

**DOI:** 10.3390/ijms12021222

**Published:** 2011-02-18

**Authors:** Kang Liu, Zhen Li, Tao Wu, Suju Ding

**Affiliations:** 1 Department of Neurology, Changhai Hospital, Second Military Medical University, Shanghai 200433, China; E-Mails: lllkkkjjj12345@sina.com (K.L.); wutao.doctor@hotmail.com (T.W.); jxleezhen@163.com (Z.L.); 2 Department of Neurology, the 456th Hospital of PLA, Jinan 250031, China

**Keywords:** ischemia reperfusion, MMP9, blood brain barrier, Laminin, Rho kinase

## Abstract

Rho kinase (ROCK) is a well-known downstream effector of Rho and plays an important role in various physiopathological processes. In this study, we aim to investigate the correlation between ROCK and microvascular damage in rat brain subjected to middle cerebral artery occlusion (MCAO) and reperfusion, and to elucidate the mechanisms underlying the microvascular damage. ROCK and matrix metalloproteinase 9 (MMP9) mRNA levels were determined by real time quantitative PCR, Laminin was detected by immunofluorescence and Blood Brain Barrier (BBB) permeability was examined by Evans Blue (EB) in rat MCAO models. We observed similar patterns of changes in ROCK expression, brain EB content, and Laminin expression at different time points after brain ischemia. Statistical analysis further confirmed a significant linear correlation of ROCK expression with the onset of microvascular damage in brain. Furthermore, the ROCK inhibitor fasudil decreased brain EB content but increased Laminin expression. These results provide strong evidence that ROCK mediates microvascular damage. In addition, we found that fasudil could significantly inhibit MMP9 expression induced by ischemia. Thus, our findings suggest that ROCK promotes microvascular damage by upregulating MMP9 and reveal ROCK as a promising therapeutic target for stroke.

## 1. Introduction

Approximately 700,000 individuals are afflicted with a stroke each year in the United States and currently approximately two million survivors of stroke live in the U.S. with prolonged disability. In China, 1.5 million people die from stroke each year and in developed countries stroke is the third leading cause of death, only surpassed by heart disease and cancer [1]. The economic, social and psychological costs of stroke are so significant that intense research has focused on understanding the underlying pathophysiology of stroke and the development of effective therapeutic options.

Therapies that reinstate cerebral blood flow (CBF) to the ischemic territory are partially efficacious but may lead to disability due to lack of neurovascular unit protection and the resulting brain microvascular damage after reperfusion. Microvascular damage, in turn, results in the accumulation of neutrophils, the activation of leukocytes and the release of inflammatory cytokines, which then promote a cellular inflammatory response and form a positive feedback. Consequently, release of oxidants, proteolytic enzymes and inflammatory cytokines change the blood brain barrier (BBB) permeability properties, leading to brain edema formation. Remarkably, matrix metalloproteinases (MMPs) released by activated leukocytes affect the integrity of the neurovascular unit, degrading the basal membrane [2]. In particular, MMP9 has been known to contribute to hemorrhages after stroke and thrombolysis by t-PA treatment [3]. Moreover, MMP9 knockout mice did not display BBB disruption after transient ischemia [4]. These results suggest that MMP9 plays an important role in BBB disruption after stroke and indicate MMP9 as a promising therapeutic target for stroke.

Rho kinase (ROCK), a downstream effector of Rho, is a serine/threonine kinase that is activated when bound to the active GTP-bound form of Rho. Recent studies demonstrated that ROCK is activated in cerebral ischemia and ROCK inhibitors reduce infarct size in focal cerebral ischemia [5]. However, the molecular details of the contribution of ROCK to brain microvascular damage remain largely elusive. Therefore, in this study, we investigated the relationship between ROCK expression and microvascular damage in rat brain subjected to middle cerebral artery occlusion and reperfusion (MCAO). We further examined the effect of ROCK inhibitor on microvascular damage and MMP9 expression to elucidate the mechanisms underlying the microvascular damage. Our results established a correlation among ROCK, brain EB content, laminin expression and MMP9 expression following MCAO, demonstrating the protective effects of fasudil on microvascular damage during cerebral ischemia.

## 2. Materials and Methods

### 2.1. Experimental Animals and MCAO Model

Adult male Sprague-Dawley rats (300 ± 30 g) were cared for according to the Guide for the Care and Use of Laboratory Animals. The committee for experimental animals of Second Military Medical University approved all surgical procedures. Rats were anesthetized with chloral hydrate (350 mg/kg intraperitoneally; IP) and subjected to MCAO as described previously [6]. In brief, we exposed the right common carotid artery, internal carotid artery, and external carotid artery surgically. A 4–0 monofilament nylon suture (Beijing Sunbio Biotech Co. Ltd.) with a rounded tip was inserted into the internal carotid artery through the external carotid artery stump and gently advanced to occlude the MCA. After 60 min of MCAO, the suture was removed to restore blood flow (reperfusion confirmed by laser Doppler). Sham-operated rats were manipulated in the same way; the monofilament was inserted to a depth of 1.0–2.0 mm with the MCA not occluded. Core body temperatures were monitored with a rectal probe and maintained at 37 °C during the whole procedure. All surgical procedures were performed under an operating stereomicroscope.

In one set, 120 rats were randomly divided into sham group, reperfusion 6 h group, reperfusion 24 h group, reperfusion 48 h group, and reperfusion 72 h group (24 rats per group). In another set, 72 rats were randomly divided into sham group, reperfusion 24 h group, and ROCK inhibitor (fasudil) treated group (24 rats per group) and were IP administered with saline or fasudil (15 mg/kg) once before operation and once 12 h after operation.

### 2.2. Ischemic Core and Penumbra Dissections

The ischemic core and penumbra of the cortex were dissected following the protocols described previously [7]. Briefly, each hemisphere was cut longitudinally, from dorsal to ventral at 1.5 mm from the midline to exclude medial brain structures that were supplied primarily by the anterior cerebral artery. A transverse diagonal incision at approximately the “2 o’clock” position separated the core from the penumbra.

### 2.3. Determination of BBB Permeability

Evans Blue (EB, 2% in saline, 4 mL/kg) was injected intravenously 24 h after the onset of MCAO. The chest was subsequently opened under halothane anesthesia 1 h later. Rats were perfused with saline through the left ventricle at 110 mm Hg pressure until colorless perfusion fluid was obtained from the right atrium. After decapitation, the brain was blocked into 2 segments that included the levels bregma +2.7 and −0.3 mm. Coronal blocks were next divided into right and left hemispheres for local measurement of EB dye. Samples were weighed and placed in 50% trichloroacetic acid solution. Following homogenization and centrifugation, the extracted dye was diluted with ethanol (1:3), and its fluorescence was determined at 620 nm. Calculations were based on external standards in the same solvent (100–500 ng/mL). The tissue content of EB was quantified from a linear standard curve derived from known amounts of the dye and was expressed per gram of tissue.

### 2.4. Immunofluorescence Staining for Laminin

For tissue harvesting, the rats were anesthetized with a lethal dose of sodium pentobarbital (80 mg/kg body weight). After decapitation, the brain was washed in 20% phosphate-buffered sucrose, then embedded and mounted in Tissue-Tek OCT compound. Serial longitudinal frozen sections (4 μm) were cut, fixed, and washed with phosphate-buffered saline (PBS) for 5 min, 3 times. After incubation with 5% normal goat serum for 1 h at RT, the sections were incubated with Laminin primary antibody (rabbit anti rat, Abcam) in a humid chamber at 32 °C for 1 h. The immunoreaction was visualized by treatment with CY3-conjugated goat anti-rabbit secondary antibody (Jackson) at 32 °C for 30 min in the dark. Immunostained sections were mounted and analyzed using a Leica epifluorescence microscope (Leica Microsystems Wetzlar GmbH, Germany). Three fields were randomly selected and semi-quantitation of the positive staining was performed using Leica-Qwin software with the florescence value calculated as Ga (positive gray value)–GA (background gray value).

### 2.5. RNA Isolation and Real-Time Quantitative PCR

Total RNA was isolated from right cortical samples by Trizol reagent (Invitrogen), and RT was performed with TOYOBO RT kit (TOYOBO) according to the manufacturer’s instructions. PCR was performed with primers for ROCK (forward: 5′-CTGCTGAAGTCGTGCTTGCA-3′;

Reverse: 5′-AGCATGTTATCGGGCTTCACA-3′, amplicon: 79 bp);

MMP9 (forward: 5′-ACGAGGACTCCCCTCTGCAT-3′;

Reverse: 5′-AGGCCTTGGGTCAGGTTTAGA-3′, amplicon: 83 bp);

GAPDH was used as an internal standard (forward: 5′-CAGTGCCAGCCTCGTCTCAT-3′;

Reverse: 5′-TGGTAACCAGGCGTCCGATA-3′, amplicon: 79 bp).

The PCR products were quantified by ABI Prism^®^ 7900HT Sequence Detection System.

### 2.6. Statistical Analyses

All data are expressed as *χ̄* ± *s*. All data were analyzed with SPSS11.0 statistical software. One-way ANOVA, Bonferroni, Kruskal-Wallis H test and Pearson correlated analysis were performed. *P* < 0.05 was considered as statistically significant.

## 3. Results

### 3.1. Expression of ROCK at Different Time Points after MCAO

By real-time quantitative PCR, we observed the changes of mRNA level of ROCK at different time points after MCAO (Table 1). The expression level of ROCK increased 6 h after reperfusion and the increase was significant compared with a sham group (*P* < 0.01). The expression of ROCK kept increasing 6–24 h after reperfusion, and then decreased gradually 48 h after perfusion. The different time points group all showed significant difference in ROCK expression compared with the sham group (*P* < 0.01), while there was no significant difference between 24 h after reperfusion group and 6 h after reperfusion group (*P* > 0.05).

### 3.2. BBB Permeability at Different Time Points after MCAO

By EB method, we measured EB contents at different time points after MCAO (Table 1). Clearly, EB content increased 6 h after perfusion, with significant difference from sham group (*P* < 0.01). EB content gradually increased 6–48 h after perfusion, suggesting that BBB permeability increased gradually and microvascular structure was gradually damaged. However, EB content began to decrease 48 h after perfusion. The difference in EB content was significant when each individual group was compared with sham group (*P* < 0.01) or compared with each other (*P* < 0.01).

### 3.3. Expression of Laminin at Different Time Points after MCAO

**Figure 1.**
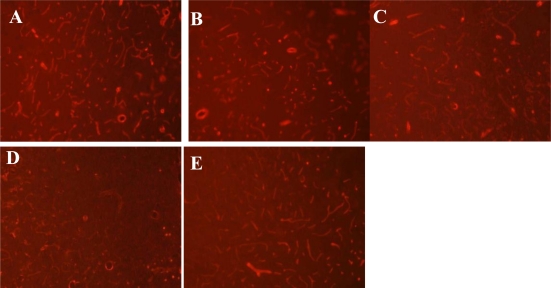
Immunofluorescence staining for Laminin at basal membrane in rat ischemic penumbra. Laminin expression was observed using aLeica epifluorescence microscope. The red signal represents positive staining. (**A**) sham; (**B**) reperfusion 6 h; (**C**) reperfusion 24 h; (**D**) reperfusion 48 h; (**E**) reperfusion 72 h. Amplification: 40×.

By immunofluorescence staining, we detected the expression of Laminin in the microvascular basal membrane in rat ischemic penumbra. As shown in Figure 1, we observed different staining patterns for Laminin at different time points after MCAO. While strong positive staining for Laminin was detected in the sham group, slightly weaker staining was observed 6 h after perfusion. Moreover, 24 h after perfusion, the staining for Laminin was significantly weaker with both the positive areas and the staining intensity decreased. We could also observe basal membrane damage such as breakdown. The weakest staining for Laminin was observed at 48 h after perfusion, when both the positive areas and staining intensity were lowest. The staining was blurry and the shape was irregular, demonstrating that substantial basal membrane damage occurred. In comparison, the staining for Laminin was recovered and microvascular regeneration could be observed 72 h after perfusion.

To obtain a quantitative comparison of Laminin expression, we performed semi-quantitative analysis with Leica-Qwin software. The results demonstrated that Laminin expression decreased gradually 6–48 h after perfusion, was lowest at 48 h after perfusion, and then gradually increased (Table 1). At the different time points, all groups showed significant difference in Laminin expression compared with the sham group (*P* < 0.01). In addition, although there was no significant difference between the 24 h after reperfusion group and 72 h after reperfusion group (*P* > 0.05), there was a significant difference when any other two groups were compared (*P* < 0.05).

### 3.4. Correlation among ROCK mRNA Level, Brain EB Content and Laminin Expression after MCAO

Since we observed similar changes in ROCK mRNA level, brain EB content and Laminin expression after MCAO, next we attempted to address their association. Based on statistical analysis, the correlation coefficient r between ROCK expression and brain EB content was 0.925, and it was statistically significant (*P* = 0.025 < 0.05). The correlation coefficient r between ROCK expression and Laminin expression was −0.955, and it was also statistically significant (*P* = 0.011 < 0.05). Thus, these data suggest that ROCK expression is positively correlated with BBB permeability and negatively correlated with Laminin expression.

### 3.5. ROCK Inhibitor Decreases BBB Permeability after MCAO

Since ROCK plays a role in the pathology of stroke, we decided to use the ROCK inhibitor fasudil to evaluate its effects on microvascular damage after MCAO. First, we examined the impact of fasudil on BBB permeability by measuring brain EB content. As shown in Table 2, compared with the sham group, brain EB content was significantly higher in both the 24 h reperfusion group and 24 h reperfusion plus fasudil group (*P* < 0.05). However, brain EB content was significantly lower in the 24 h reperfusion plus fasudil group than in the 24 h reperfusion group (*P* < 0.05), suggesting that the ROCK inhibitor decreases BBB permeability.

### 3.6. ROCK Inhibitor Rescues Laminin Expression after MCAO

**Figure 2.**
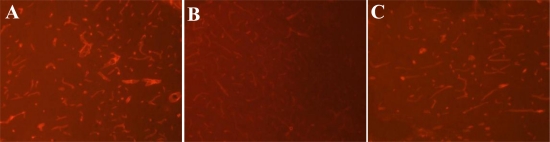
Immunofluorescence staining for Laminin at basal membrane in rat ischemic penumbra. Laminin expression was observed using a Leica epifluorescence microscope. The red signal represents positive staining. (**A**) sham; (**B**) reperfusion 24 h; (**C**) reperfusion 24 h + fasudil. Amplification: 40 ×.

Next we examined the effect of fasudil on basal membrane by immunofluorescence staining for Laminin. As shown in Figure 2, compared with the sham group, 24 h after perfusion, the staining for Laminin was much weaker and basal membrane breakdown was obvious. Interestingly, fasudil partially rescued positive staining for Laminin and promoted microvascular regeneration.

Further semi-quantitative analysis demonstrated that Laminin expression was significantly lower in both the 24 h reperfusion group and 24 h reperfusion plus fasudil group compared with the sham group (*P* < 0.05). Nevertheless, Laminin expression was significantly higher in the 24 h reperfusion plus fasudil group compared with the 24 h reperfusion group (*P* < 0.05) (Table 2). Collectively, these data suggest that the ROCK inhibitor could antagonize basal lamina damage and the downregulation of Laminin following brain ischemia reperfusion.

### 3.7. ROCK Inhibitor Inhibits MMP9 Expression after MCAO

Finally, we examined the effect of fasudil on MMP9 expression after MCAO. Real-time quantitative PCR experiments demonstrated that the mRNA level of MMP9 was significantly higher in both the 24 h reperfusion group and the 24 h reperfusion plus fasudil group, compared with the sham group (*P* < 0.05). However, the mRNA level of MMP9 was significantly lower in the 24 h reperfusion plus fasudil group compared with 24 h reperfusion group (*P* < 0.05) (Table 2). Thus, these results indicate that ROCK inhibitor inhibits MMP9 expression following brain ischemia reperfusion.

## 4. Discussion

Cerebral microvessels have a unique feature, which protects sensitive brain cells from disturbing elements circulating in the blood. The BBB exists primarily as a selective diffusion barrier at the level of the cerebral microvascular endothelium, characterized by the presence of tight cell-cell junctions and a lack of fenestrations. Pericytes and endothelial cells are ensheathed by the basal membrane, a 30 to 40 nm thick membrane. The basal membrane is one of the three basic components in the microvasculature and its main component is Laminin. Accumulating evidence indicates that Laminin dysfunction leads to basal membrane damage, microvascular impairment and BBB defects, which in turn results in brain edema and hemorrhage transformation after perfusion. It has been reported recently that Laminin expression was downregulated during ischemia-reperfusion [8]. Thus, Laminin level may reflect the integrity of basal membrane and indicate possible microvascular damages.

ROCK is a well-known downstream effector of Rho and plays an important role in various physiopathological processes. For example, ROCK is implicated in vascular smooth muscle cell contraction, actin cytoskeleton organization, cell adhesion and motility, and gene expression. At the molecular level, ROCK activates signaling cascades that accelerate inflammation/oxidative stress, thrombus formation, and fibrosis, thus contributing to microvascular damage.

Recently, the ROCK inhibitor fasudil was reported to protect the vascular endothelium by inhibiting neutrophil adhesion and reducing neutrophil-induced endothelial injury, indicating the critical role of the Rho/ROCK pathway in diabetic retinal microvasculopathy [9]. In the present study, we found similar pattern of changes in ROCK expression, brain EB content that indicates BBB permeability, and Laminin expression at different time points after brain ischemia reperfusion. Statistical analysis further confirmed the correlation among ROCK mRNA level, brain EB content and Laminin expression as we documented. These results strongly suggest that ROCK is involved in microvascular damage. Furthermore, by use of the ROCK inhibitor fasudil, we found that fasudil decreased brain EB content but increased Laminin expression, thus exhibiting protective effects against microvascular damage. These results provide further evidence that ROCK mediates microvascular damage.

Matrix metalloproteinases (MMPs) play important roles in the pathogenesis of CNS diseases that share common pathophysiological processes, such as BBB disruption, oxidative stress, remodeling of the extracellular matrix (ECM) and inflammation [10]. The expression of MMPs, especially MMP2, MMP9 and MMP10, is induced by cerebral ischemia [11]. Among MMPs, MMP9 is crucially involved in BBB disruption due to the degradation of the ECM and damage of basal lamina, which consequently leads to brain edema and hemorrhage [12–14]. Most recently, MMP9 was found to promote hemorrhagic transformation and spontaneous intracerebral hemorrhage during hypertension [15]. MMP9 is also involved in the pathogenesis of early brain injury of subarachnoid hemorrhage through degrading Laminin [16]. In accordance with these reported studies, here we found that MMP9 expression was increased significantly in the rat ischemic penumbra 24 h after reperfusion, thus confirming the induction of MMP9 by brain ischemia. Interestingly, we found that fasudil could significantly inhibit MMP9 expression induced by ischemia, suggesting that ROCK may promote microvascular damage by upregulating MMP9 expression during ischemia reperfusion. However, further investigation of the potential mechanism by which ROCK regulates MMP9 during cerebral ischemia will be necessary to confirm our conclusion and will be the aim of our next study.

In summary, to our knowledge, we are the first to establish the correlation among ROCK mRNA level, brain EB content and Laminin expression following MCAO. Our results provide strong evidence that ROCK mediates microvascular damage. In addition, by use of the ROCK inhibitor fasudil, our study suggests a novel mechanism by which ROCK may upregulate MMP9 expression to contribute to microvascular damage during cerebral ischemia. Taken together, our findings reveal ROCK as a promising therapeutic target for stroke.

## Figures and Tables

**Table 1.** The mRNA level of ROCK, brain Evans Blue (EB) content, and semi-quantitation of Laminin expression following middle cerebral artery occlusion (MCAO).

**Group**	***n***	**ROCK mRNA**	**EB content (μg/g)**	**Laminin expression**
sham	5	0.466 ± 0. 030	0.202 ±0.019	168.91 ± 6.73
ischemia reperfusion				
reperfusion 6 h	5	0.617 ± 0.017 *	1.372 ± 0.123 *	149.45 ± 4.38 *
reperfusion 24 h	5	0.662 ± 0.016 *	3.764 ± 0.160 *	115.56 ± 8.29 *
reperfusion 48 h	5	0.838 ± 0.036 *	4.650 ± 0.286 *	82.95 ± 12.33 *
reperfusion 72 h	5	0.713 ± 0.018 *	2.982 ± 0.163 *	124.34 ± 5.29 *

* *P* < 0.01 *vs.* sham group.

**Table 2.** The mRNA level of MMP9, brain Evans Blue (EB) content, and semi-quantitation of Laminin expression following middle cerebral artery occlusion (MCAO) and fasudil treatment.

**Group**	**EB content (mg/g) (*n* = 5)**	**Laminin expression (*n* = 5)**	**MMP9 mRNA (*n* = 10)**
sham	0.210 ± 0.039	167.89 ± 8.97	0.514 ± 0.055
MCAO	3.376 ± 0. 134 *	109.72 ± 12.01 *	0.762 ± 0.021 *
Fasudil	1.930 ± 0.079 *^,^†	142.02 ± 4.82 *^,^†	0.659 ± 0.028 *^,^†

* *P* < 0.05 *vs.* sham group;

† *P* < 0.05 *vs.* MCAO group.
